# Anti-adsorption Mechanism of Photoresist by Pluronic Surfactants: An Insight into Their Adsorbed Structure

**DOI:** 10.1021/acs.langmuir.3c00714

**Published:** 2023-05-20

**Authors:** Masaki Hanzawa, Taku Ogura, Koji Tsuchiya, Masaaki Akamatsu, Kenichi Sakai, Hideki Sakai

**Affiliations:** † NIKKOL GROUP Nikko Chemicals Co., Ltd., 3-24-3 Hasune, Itabashi, Tokyo 174-0046, Japan; ‡ Research Institute for Science and Technology, Tokyo University of Science, 2641 Yamazaki, Noda, Chiba 278-8510, Japan; § Department of Chemistry and Biotechnology, Faculty of Engineering, Tottori University, 4-101 Koyama-Minami, Tottori 680-8552, Japan; ∥ Department of Pure and Applied Chemistry, Faculty of Science and Technology, Tokyo University of Science, 2641 Yamazaki, Noda, Chiba 278-8510, Japan

## Abstract

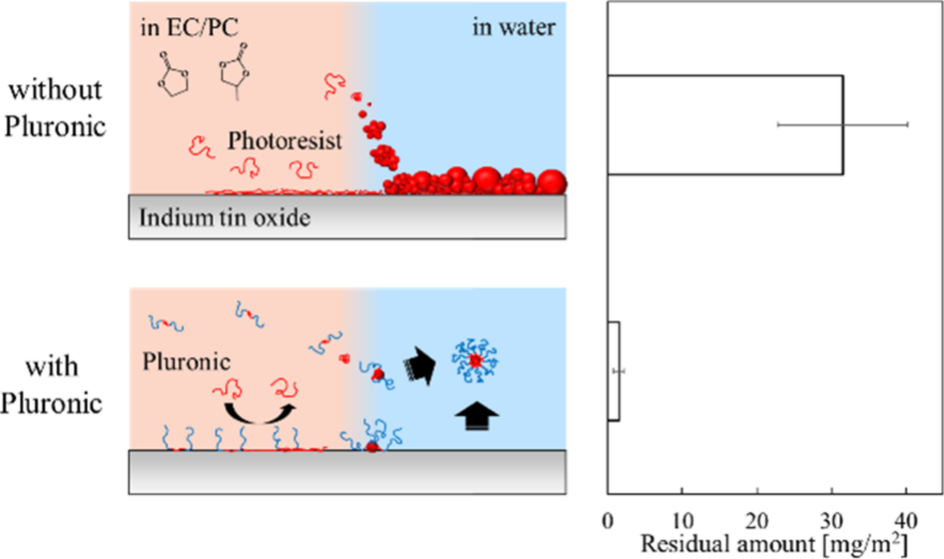

Photoresist stripping is the final step in the photolithography process that forms fine patterns for electronic devices. Recently, a mixture of ethylene carbonate (EC) and propylene carbonate (PC) has attracted attention as a new stripper based on its eco-friendliness and anti-corrosiveness. However, the EC/PC mixture causes re-adsorption of the photoresist during a process of subsequent water rinsing. In this study, we characterized the adsorption/desorption of the photoresist and a triblock Pluronic surfactant [poly(ethylene oxide)–poly(propylene oxide)–poly(ethylene oxide)] as a blocking agent on an indium tin oxide (ITO) substrate. In addition, we evaluated the dispersion of photoresist particles. The photoresist polymer formed a thin and rigid adsorption layer on an ITO substrate in the EC/PC mixture. When water was injected into the EC/PC mixture and the photoresist solutions, the photoresist polymer aggregated and was then deposited on the substrate. In contrast, the addition of Pluronic surfactant F-68 (PEO_79_PPO_30_PEO_79_) into the EC/PC mixture remarkably decreased the residual amount of the photoresist on the ITO after water injection. This variation was attributed to the PEO blocks of F-68 extended to the solution phase, whereas the PPO blocks of F-68 functioned as anchors for adsorption onto the photoresist. Therefore, the F-68-adsorbed layer prevented interaction between the photoresist particles or the photoresist and the ITO surface, which provides potential for future applications as new stripping agents with high removal performance.

## Introduction

Photoresists are photofunctional materials used in the electronics industry. Photoresist films are mainly composed of polymers and photosensitizers and possess both etching resistance and light-induced developer solubility. These films are widely known as chemical agents for forming fine metal patterns in applications such as liquid crystal displays, semiconductor devices, microelectromechanical systems, and integrated circuits.^1−4^ Typically, a photoresist film is stripped quickly and completely from the substrate after etching, as the residual photoresist can cause wiring abnormalities or disconnections in subsequent manufacturing processes. Amine-type agents, such as monoethanolamine and *N*-methylpyrrolidone, are often used as strippers for photoresist films; however, these agents may damage the metal surface.^5,6^ Hence, alternatives to these reagents are required to avoid potential damage to the metal surface.

Alkylene carbonates, particularly ethylene carbonate (EC) and propylene carbonate (PC), are extensively used in many industrial applications.^6−8^ These compounds have been considered new photoresist-stripping agents because they are less toxic and corrosive to the substrate than amine-type agents.^5,6^ However, the photoresist stripped from the substrate can be readily redeposited onto the surface via water rinsing. Amphiphilic materials are typically added to prevent such unexpected adsorption.

The quartz crystal microbalance with dissipation monitoring (QCM-D) technique is highly sensitive to the real-time mass change and viscoelastic properties of adsorbed films.^9^ This technique provides information on the adsorption and desorption of target materials in water or organic solvents through sensor coating. Hence, QCM-D has been widely employed as a tool for monitoring dynamic interactions between solid and liquid interfaces. One of these applications is the non-specific adsorption of proteins or cell adhesion on surfaces. Hydrophobic surfaces or stainless steel covered with Pluronic surfactants [poly(ethylene oxide)–poly(propylene oxide)–poly(ethylene oxide); PEO_*x*_PPO_*y*_PEO_*x*_] or PEO homopolymers reportedly impede the adsorption of proteins.^10,11^ In the presence of such polymer brushes on the surfaces, the stretched polymer chains become entropically unfavorable and cause steric repulsion between the chains and proteins. The effect of the PEO-based coating on the repel adsorption is related to the grafting density and chain length.

In our previous studies, we characterized the adsorption of Pluronic surfactants with different PEO chain lengths on silica in a mixture of EC and PC using atomic force microscopy (AFM).^12^ The surfactants were dissolved in the EC/PC mixture, with the PEO and PPO chains as solvophilic and solvophobic groups, respectively; the longest PEO chain analogue (PEO_79_PPO_30_PEO_79_; F-68) formed a polymer brush on the silica surface. Furthermore, we demonstrated the dispersion state of photoresist particles and their adsorption/desorption behavior on an indium tin oxide (ITO) substrate in an EC/PC/water mixture with and without F-68.^13^ QCM-D revealed that the F-68 adsorption layer prevented aggregation between the photoresist particles and their adsorption on the ITO substrate. However, the conformation of the F-68 molecules adsorbed on the photoresist is still unclear. Particularly, the conformation or adsorption layer structure in the region of water-rich compositions is crucially important, according to the enhanced anti-adsorption ability of F-68 in such a region.^13^ In addition, there is a lack of discussion about the adsorption kinetics in organic solvents, including our EC/PC mixtures.

In this study, we monitored the adsorption and desorption processes of the photoresist on an ITO substrate in an EC/PC mixture with F-68 by QCM-D. The adsorption structure of F-68 on the photoresist in either a water/EC/PC mixture or pure water was analyzed by small angle X-ray scattering (SAXS) and AFM. Our findings on the systematic evaluation of anti-adsorption can serve as an important platform for many industries, including not only electronics but also textiles, metals, foods, and biomaterials.

## Experimental Section

### Materials

EC and PC used were obtained from Kanto Chemical (Tokyo, Japan) without further purification. The weight ratio of the EC/PC mixture was fixed at 70/30.^12^

The Pluronic surfactant [F-68 (PEO_79_PPO_30_PEO_79_)] was obtained from ADEKA (Tokyo, Japan) and used without purification. The average molar masses of the PEO and PPO chains are 6600 and 1750 g mol^–1^, respectively; the total average molar mass is 8350 g mol^–1^. The concentration of F-68 was set to 1 or 10% w/w.

The positive photoresist (AZ SR-220, AZ Electronic Materials) used in this study comprised a novolak-type phenolic resin derivative as the primary component and naphthoquinonediazide sulfonate as a secondary component. Because the photoresist material contains more than 80% w/w propylene glycol monomethylethyl ether acetate (PGMEA) as a solvent, EC, the main component of the stripper, was added, and PGMEA was removed by distillation. This solution (15% w/w in EC) was used to prepare the photoresist dispersion.

The water used in this study was filtered through a Millipore membrane filter (0.1 μm pore size) after deionization using a Barnstead NANO pure Diamond UV system.

### Preparation of Photoresist Dispersion

The photoresist dispersion was prepared according to the actual stripping processes reported in the literature.^13^ The photoresist film coated on the substrate was immersed twice in a stripping agent at 80 °C, then immersed once in the same stripping agent at 45 °C, and finally rinsed with pure water at room temperature. Considering these processes, we prepared a photoresist dispersion by adding an EC-distilled photoresist solution to the stripping agents (EC/PC solvent and EC/PC/F-68 solution) at 80 °C, incubating at 45 °C, and then adding water. The weight ratio of EC/PC-to-water in the dispersion was set to 25/75, where the hydrophobic photoresist dispersion was visually clear in the presence of F-68 whereas turbid in the absence of F-68.^13^ The concentration of the photoresist was fixed at 0.1% w/w. These dispersions were evaluated using freeze-fracture transmission electron microscopy (FF-TEM) and SAXS.

### Formation of the Photoresist Film

An ITO-coated QCM-D sensor (Biolin Scientific, QSX 999) was sonicated in both ethanol and pure water. The substrate was dried under N_2_ gas and cleaned by ultraviolet (UV) irradiation using a BioForce Nanosciences UV/ozone ProCleaner to remove organic contaminants. The photoresist film was formed according to the following procedure: (i) the photoresist/PGMEA solution was dropped onto the surface in approximately 20 μL; (ii) the substrate was rotated by a spin-coater (SC-200, Oshigane Co., Ltd.) with an initial speed of 500 rpm (10 s) and then at 3000 rpm (30 s) to form a thin film; and (iii) the solvent was removed by heating for 5 min in a thermostatic bath (FO-60W, Tokyo Garasu Kikai Co., Ltd.) set at 130 °C. Preliminary spectroscopic ellipsometry experiments (FS-1, Film Sense) revealed that the thickness of the photoresist film was 440–450 nm.^14^ The photoresist-coated ITO sensor was used for AFM measurements.

### QCM-D Measurements

QCM-D measurements were performed using a Biolin Scientific QSense Explorer instrument. The relationship between the shift in frequency (Δ*F*_*n*_) and the adsorption amount (Δ*m*) for the thin and rigid layers is approximated by the Sauerbrey equation (eq 1).^15^
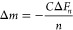
1where *C* is the mass sensitivity constant [*C* = 0.177 mg/(Hz·m^2^) for a 5 MHz crystal] and *n* is the overtone number. The energy dissipation shift Δ*D*_*n*_ is obtained simultaneously with the frequency shift and is defined according to eq 2.
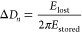
2where *E*_lost_ is the dissipated energy and *E*_stored_ is the total energy stored in the oscillator during an oscillation cycle. The liquid flow rate was maintained at 0.1 mL/min, and the selected overtone numbers (*n*) were 5, 7, and 9. All the experiments were conducted at 25 °C.

For thick and soft films, the QCM-D profiles exhibited a high energy dissipation shift and overtone number (frequency) dependence. In this study, we applied the widely used Voigt model,^16^ which enables the estimation of film thickness (*d*), shear elastic modulus (μ), and shear viscosity (η) by fitting the experimental data for Δ*F*_*n*_ (eq 3) and Δ*D*_*n*_ (eq 4).
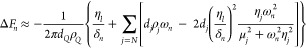
3
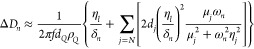
4where ρ, δ, and ω are the density, viscous penetration depth ((2η_*l*_/(ρ_*l*_·ω))^1/2^), and 2π*f*, respectively; the subscripts *Q*, *l*, and *j* represent the quartz crystal, liquid medium, and adsorbed film, respectively. *N* is the number of the viscoelastic overlayers (assumed to be 1). Both viscoelastic parameters, μ and η, can be related to the complex elastic modulus (G*) through eq 5.^17^
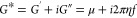
5where *G*′ and *G*″ are the storage and loss modulus of the adsorbed film, respectively; the ratio of *G*″/*G*′ allowed quantification of the viscoelastic properties of the film.

### FF-TEM Analysis

FF-TEM analysis was performed using a Hitachi High-Tech H-7650 instrument to observe the photoresist dispersion state in bulk. The samples were rapidly frozen in liquid propane (<−170 °C) using a quick freezer (EM CPC, Leica) and cut with a glass knife. The replica was prepared by exposing the cross-section of the frozen dispersion to platinum vapor, followed by treatment with carbon vapor to build the replica using a freeze-replica apparatus (FR-7000A, Hitachi High-Tech). The replica was washed with a chloroform/methanol (v/v = 2/1) solution, acetone, and water. The replica was visualized at an acceleration voltage of 100 kV.

### SAXS Measurements

SAXS measurements were performed using a Xenocs Xeuss 3.0 instrument to evaluate the structure of the photoresist dispersion. The sample was sealed in a quartz capillary with an inner diameter of 1.5 mm and irradiated with Cu Kα X-ray (wavelength of 0.154 nm) from a camera length of 1000 mm for 10 min. Scattered X-rays were collected using a detector (Pilatus R 100 K, DECTRIS). The beam had a diameter of 400 μm (high-resolution mode). The obtained two-dimensional scattering pattern was circularly averaged and converted into one-dimensional (1D) data (scattered vector range of 0.01 < *q* < 2 nm^–1^). Background subtraction was performed on the 1D data. These conversions and background subtractions were calculated using the XSACT software. All measurements were conducted at room temperature.

The scattering profiles were further analyzed using the indirect Fourier transformation (IFT) method.^18,19^ The intensity can be described according to eq 6.
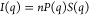
6where *I*(*q*) is the scattering intensity, *n* is the number density of the particles, *P*(*q*) is the form factor providing information on the particle size and shape, and *S*(*q*) is the structure factor representing the particle interaction potential. In this study, the coherent scattering between particles was negligible [*S*(*q*) = 1]^20^ because of the diluted photoresist systems (0.1% w/w), whereas the intensity was proportional to *P*(*q*) (eq 7).
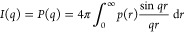
7where *p*(*r*) is the pair-distance distribution function (PDDF) and *r* is the distance between two scattering centers within the particle. The PDDF is a histogram of the distance within the scattering particles, converging to a zero level beyond the maximum diameter within the particle. As the PDDF also depends on the electron density inside the particle, the theoretical curve reflects the particle size, shape, and internal structure. Furthermore, the electron density profile [Δρ(*r*)] was calculated from the PDDF using the deconvolution method. Thus, Δρ(*r*) yields information on the size of the domains at a distance (*r*) from the center of the particle.

### AFM Analysis

AFM analysis was performed using a Hitachi High-Tech AFM 5200S/5200II instrument to evaluate the adsorption structure of F-68 on the photoresist film in water. We used triangular silicon nitride cantilevers (OMCL-TR800PSA, Olympus); the cleaning of the cantilevers was carried out according to a previous study.^12^ The force curves were obtained in an F-68 aqueous solution (1% w/w) and then measured similarly in water after repeated replacement with pure water 10 times. All experiments were conducted 10 min after the immersion of the ITO sensor at room temperature.

## Results and Discussion

### Re-adsorption of Photoresist without Pluronic Surfactant F-68

**Figure 1:**
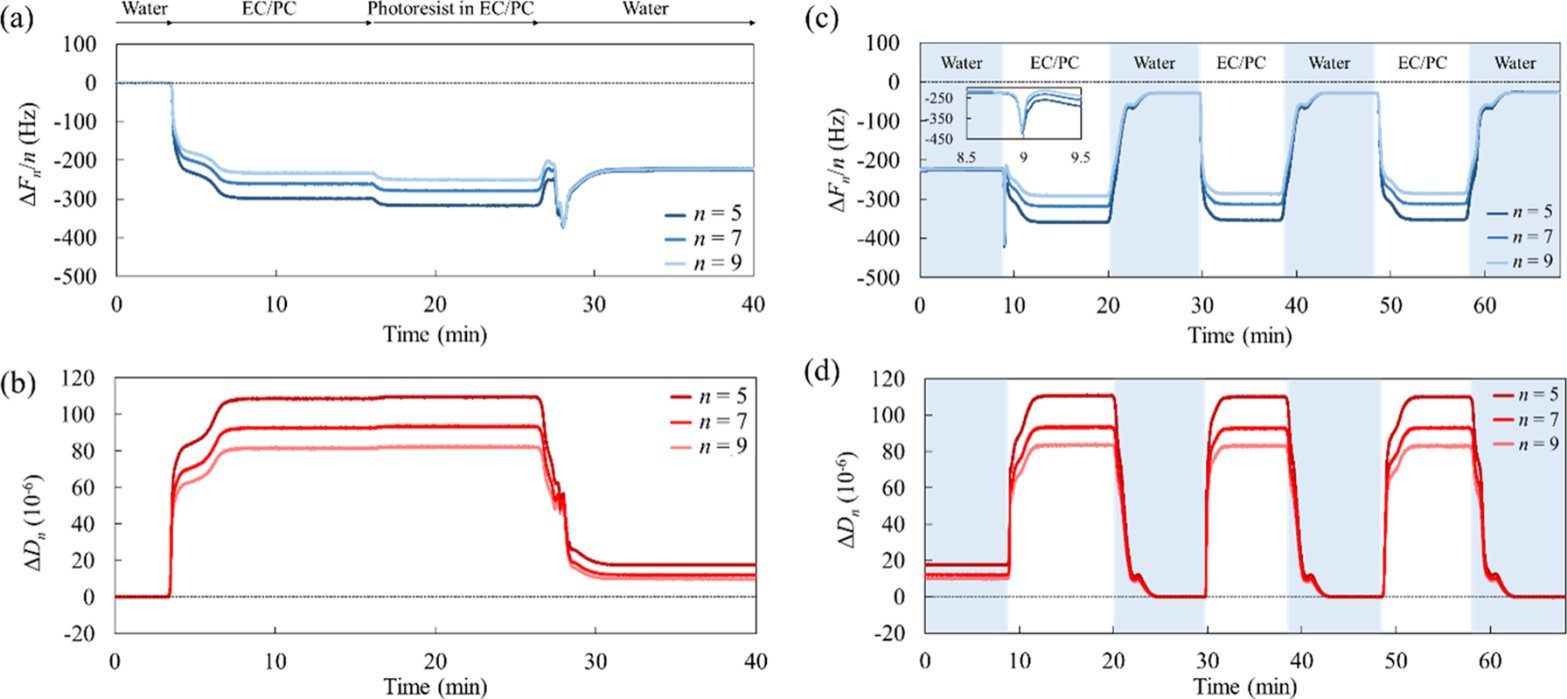
(a,c) Frequency and (b,d) dissipation shifts as a function of time of the 5th, 7th, and 9th overtones: (a,b) the adsorption of the photoresist on the bare ITO substrate, and (c,d) the repeated removal of the photoresist films (inset: enlarged view of the Δ*F*_*n*_/*n* just after the injection of the EC/PC mixture).

Before examining the prevention of the re-adsorption of the photoresist for the Pluronic surfactant, the adsorption and desorption behaviors without additives must be understood. The QCM-D results obtained in the absence of Pluronic F-68 are shown in Figure 1a,b. Initially, water was continuously injected into the QCM-D flow module until stable baselines were obtained in the frequency and dissipation shifts. The same procedure was performed for the EC/PC mixture; water and EC/PC were miscible in any proportion. Subsequently, the photoresist solution in EC/PC (0.1% w/w) was injected, and the substrate was rinsed with water.

When water was replaced with the EC/PC mixture, Δ*F*_*n*_/*n* and Δ*D*_*n*_ decreased and increased, respectively. These shifts reflect the variation in bulk viscosity and density between liquids (the bulk effect) rather than the adsorption effect. In practice, the shifts were nearly identical to the values calculated using eqs 3 and 4 (Δ*F*_5_/5 = −273 Hz, Δ*F*_7_/7 = −231 Hz, and Δ*F*_9_/9 = −204 Hz; Δ*D*_5_ = 110 × 10^–6^, Δ*D*_7_ = 93.3 × 10^–6^, and Δ*D*_9_ = 82.3 × 10^–6^), assuming that the parameter for the adsorbed film (subscript *j*) was 0. For the above calculations, the viscosity (η_*l*_) and density (ρ_*l*_) of the EC/PC mixture were experimentally determined to be 2.42 × 10^–3^ kg/(m·s) and 1322 kg/m^3^, respectively. The discrepancy between the calculated and experimental values for Δ*F*_*n*_/*n* may result from the ITO surface roughness. Upon injecting the photoresist solution, the Δ*F*_*n*_/*n* and Δ*D*_*n*_ changed further, indicating that a photoresist adsorption layer was formed on the ITO substrate. Even after rinsing with water, the shifts did not reach the initial baseline levels. Then, we confirmed monodispersed (primary-like) and aggregated (secondary-like) particles on the ITO sensor surface after QCM-D measurements [Supporting Information, Figure S1: scanning electron microscopy (SEM) images]. The adsorption of 1–10 μm sized particles reportedly causes a positive frequency shift occasionally in the QCM-D measurement,^26^ whereas, in this case, the Δ*F*_*n*_/*n* showed negative shifts at all overtone numbers, which suggests that the photoresist particles adsorbed strongly and firmly onto the ITO substrate.

From the frequency and dissipation shifts in several overtone numbers (*n* = 5, 7, and 9), we analyzed the viscoelastic properties of the photoresist film in an EC/PC mixture based on the Voigt model.^21^ We then calculated the offset from the baseline levels in the EC/PC mixture, resulting in a photoresist film thickness of 3.3 nm, a shear elastic modulus of 12 × 10^5^ kg/(m·s^2^), a shear viscosity of 11 × 10^–3^ kg/(m·s), and a *G*″*/G*′ ratio of 0.28. The photoresist film density (ρ_*j*_) was assumed to be 1250 kg/m^3^.^22^ Considering that the film is thin and *G*″*/G*′ is less than 1, the photoresist strongly interacts with the ITO substrate and forms a rigid and elastic film. The shear elastic modulus of the photoresist film on the ITO substrate in the EC/PC solvent was significantly higher than that of high-molecular-weight materials (such as polymers, proteins, or starches) in previous studies.^21,23−25^

We investigated the removability of the residual photoresist by the EC/PC mixture. Figure 1c,d demonstrates the removal of the photoresist films that were repeatedly injected with the EC/PC mixture and water using QCM-D. The time “0 min” in Figure 1c,d corresponds to 40 min in Figure 1a,b, respectively. The baseline levels represent a bare ITO substrate in water. When water was replaced for the first time with the EC/PC mixture, the profile changed in three steps: Δ*F*_*n*_/*n* decreased by approximately 200 Hz (*t* = 9.0 min in Figure 1c), followed by an immediate increase (9.0 ≤ *t* ≤ 9.2 min), and finally, a gradual decrease (9.2 ≤ *t* min). This behavior simultaneously reflects the bulk effect and desorption of the photoresist film. With water rinsing, the Δ*F*_*n*_/*n* increased to approximately −28 Hz for all overtones, whereas Δ*D*_*n*_ almost reached the baseline levels, indicating that the photoresist remained rigid on the ITO surface. Even after the same removal procedure, the QCM-D profiles were similar, and the photoresist could not be removed using only the EC/PC mixture.

### Anti-adsorption of the Photoresist with Pluronic Surfactant F-68

**Figure 2:**
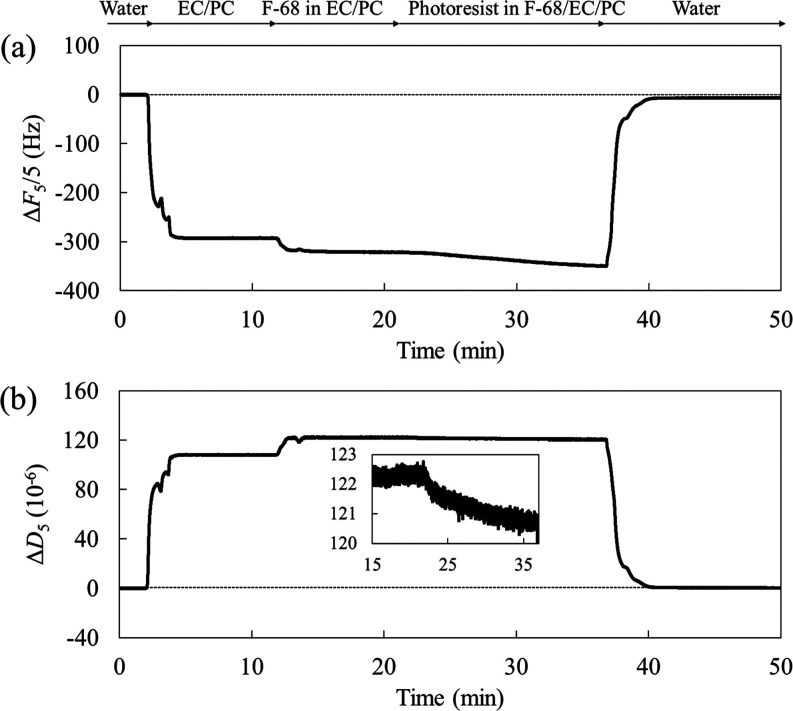
(a) Frequency and (b) dissipation shifts as a function of time of the 5th overtone for the adsorption of the photoresist with F-68 (1% w/w) on the ITO substrate (inset: enlarged view of the Δ*D*_5_ after injecting the photoresist dispersion).

The QCM-D results measured in the presence of Pluronic F-68 are shown in Figure 2. Following the procedure displayed in Figure 1a,b, we added a flow step (F-68 solution) before the F-68/photoresist solution to verify the effect of F-68. In addition, Δ*F*_*n*_/*n* and Δ*D*_*n*_ profiles were similar for all the overtone numbers. Therefore, the QCM-D results are described for *n* = 5 in the following discussion.

On replacing the F-68 and EC/PC mixture with the EC/PC mixture on the ITO substrate, Δ*F*_5_/5 decreased to −35 Hz, whereas Δ*D*_5_ increased to 20 × 10^–6^, suggesting the formation of an F-68 soft layer on the ITO substrate. In this measurement, the presence of the layer observed in the AFM profiles was also confirmed, as QCM-D could contain the bulk effect (Supporting Information, Figure S2). Although the results may include bulk effects, Pluronic surfactants have been reported to form brush structures with anchored PPO chains^12,27^ and mushroom structures.^28^ Remarkably, both Δ*F*_5_/5 and Δ*D*_5_ decreased after the injection of the F-68/photoresist solution; the Δ*D*_5_ shift reflects the detachment of F-68 from the ITO and/or the shrinking of the F-68 polymer chains. After rinsing with water, both Δ*F*_5_/5 and Δ*D*_5_ reached near-baseline levels.

**Figure 3:**
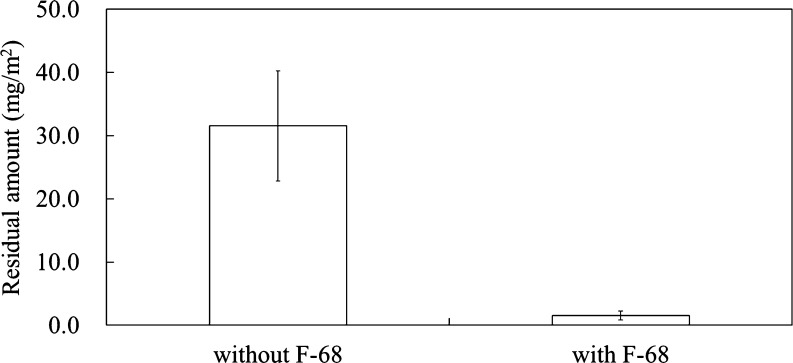
Residual amount of the photoresist on the ITO substrate after water rinsing for 10 min (standard error intervals; *N* = 4).

Figure 3 shows the residual amount of the photoresist on the ITO substrate after rinsing with water, as is shown in Figures 1a and 2a. The amount was calculated using the Sauerbrey equation (eq 1). The residue was 31.5 mg/m^2^ without F-68 and 1.53 mg/m^2^ with F-68; that is, the anti-adsorption effect was higher than 95%. In addition, the F-68 system exhibited good reproducibility. The residual amount was 4.78 mg/m^2^ after repeated rinsing with EC/PC solvent and water (Figure 1c). This value is higher than that of the system with F-68, indicating that the F-68 layer suppressed the adsorption of the photoresist, which was difficult to remove from the ITO substrate.

### Anti-adsorption Mechanism of the Photoresist by Pluronic Surfactant F-68

To obtain further insight into the anti-adsorption mechanism, we focused on (i) the anti-adsorption kinetics of the photoresist by the F-68 adsorption layer and (ii) the interaction between the photoresist and F-68 via water rinsing. These investigations have the potential to provide insights into the mechanism of the characteristic anti-adsorption.

**Figure 4:**
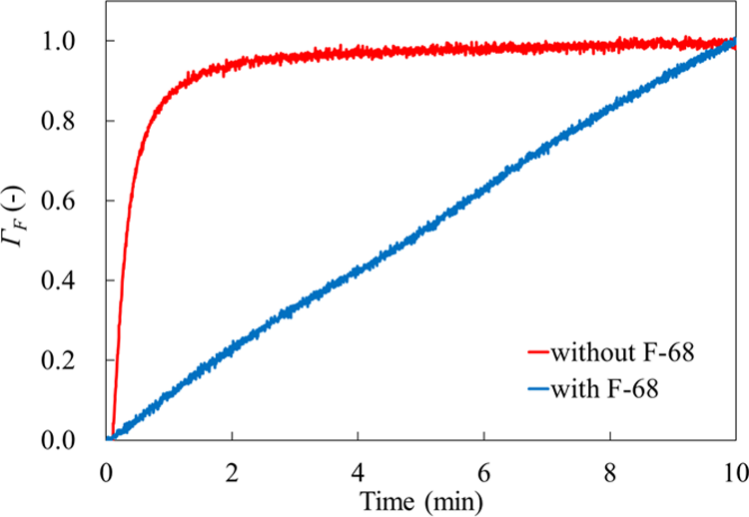
Normalized frequency shifts of the photoresist immediately after injecting the photoresist dispersion without F-68 (solid red line) and with F-68 (solid blue line) in the EC/PC mixture.

We examined the adsorption kinetics of the photoresist without F-68 (Figure 1a) and with F-68 (Figure 2a). Figure 4 shows the scaling results based on the frequency shift at 10 min after starting the injection of the photoresist dispersion according to eq 8.
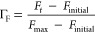
8where Γ_F_ is the normalized adsorption ratio, *F*_*t*_ is the frequency shift after a time *t*, *F*_initial_ is the frequency shift at 0 s, and *F*_max_ is the frequency shift at 10 min. In the absence of F-68, the adsorption ratio exceeded 50% in less than 30 s and reached 90% within 80 s, whereas the addition of F-68 prevented the photoresist adsorption rate. This indicates that the adsorption of the photoresist polymers is suppressed by the PEO chain of F-68 extending from the ITO surface to the bulk solution.

**Figure 5:**
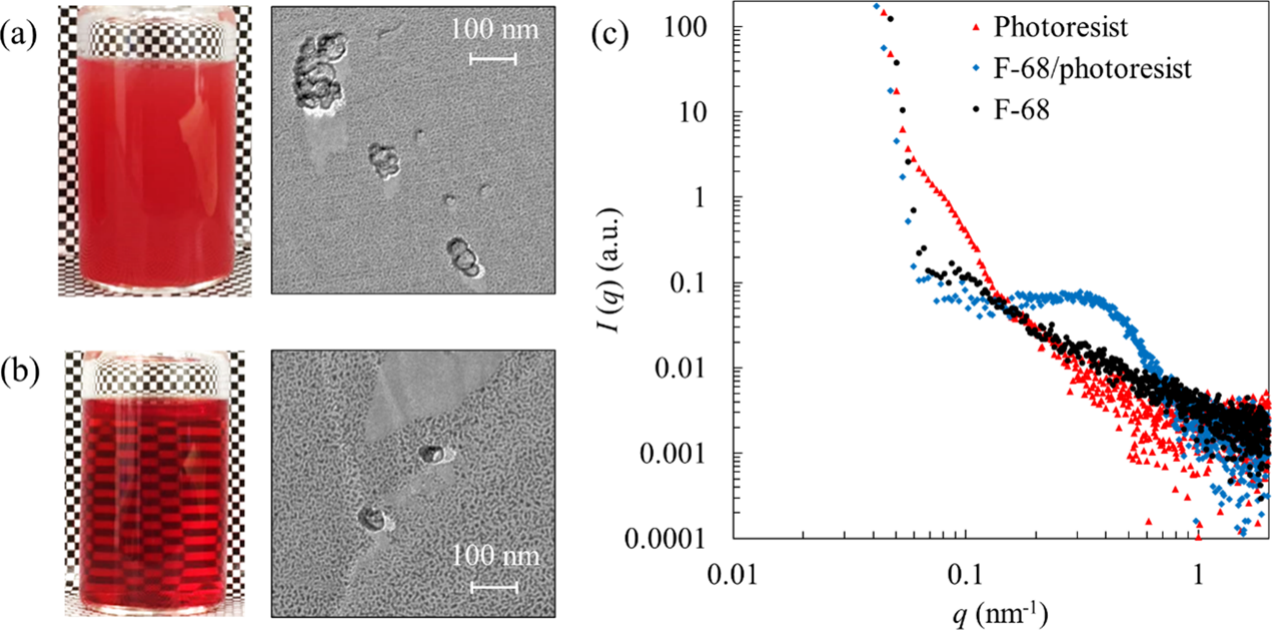
Visual representations (left) and FF-TEM (right) images of the photoresist in the mixture of EC/PC and water (a) without F-68 and (b) with 1% w/w F-68. (c) SAXS profiles of the photoresist dispersion without (red-filled triangles) and with 10% w/w F-68 (blue-filled diamonds) and 10% w/w F-68 solution (black-filled circles). The solvent weight ratio of EC/PC and water was fixed to 25/75. Then, the photoresist concentration is unified at 0.1% w/w.

**Figure 6:**
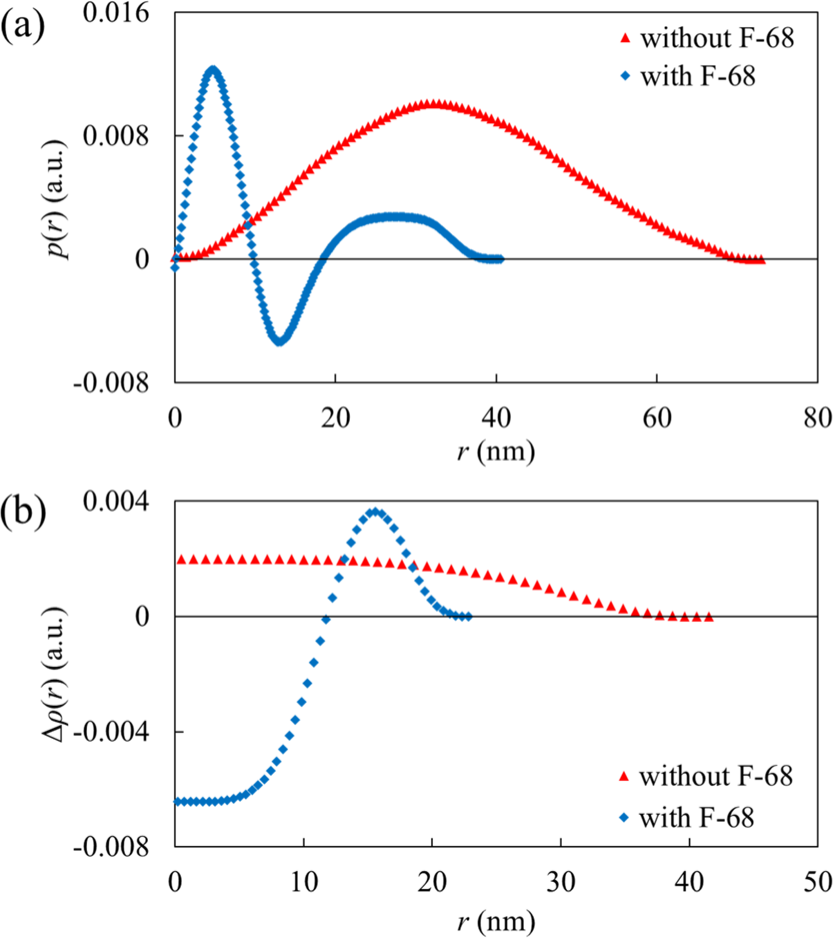
(a) PDDF and (b) electron density profiles of the photoresist dispersion without (red-filled triangles) and with (blue-filled diamonds) F-68 calculated by the IFT analysis of the SAXS intensity profiles.

**Figure 7:**
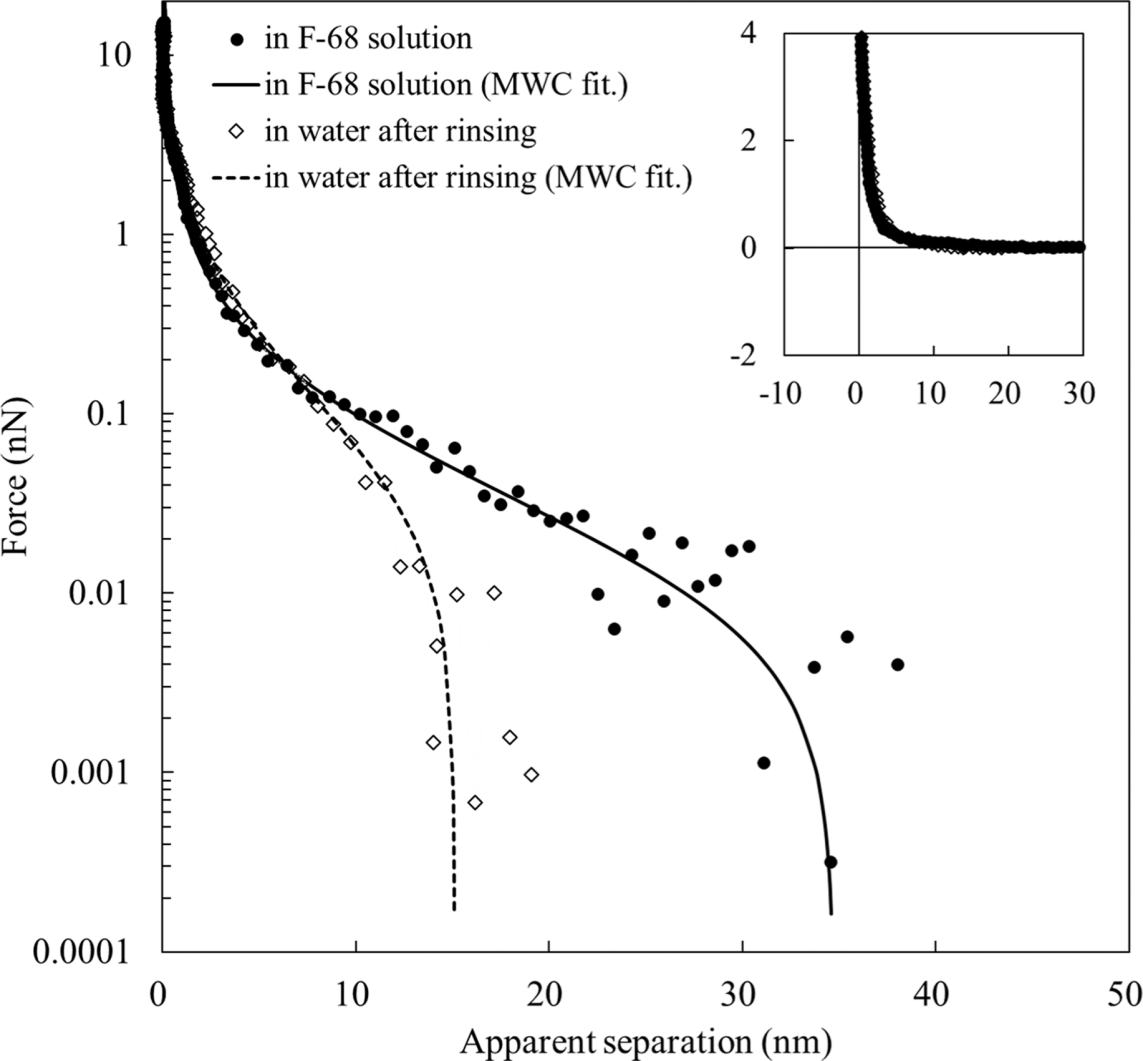
AFM approaching force curve data in a 1% w/w F-68 aqueous solution (filled markers) and in water after rinsing (open markers) on the photoresist film. The inset graph is converted to a linear scale for the *y*-axis. The solid and dashed lines represent fitting curves based on the MWC model corresponding to the experimental data in F-68 aqueous solution and water, respectively.

We analyzed the structure of F-68 adsorbed on the photoresist by means of FF-TEM and SAXS in a mixture of water/EC/PC (Figures 5 and 6) and AFM in an aqueous solution (Figure 7). Visual representations and FF-TEM images of the photoresist dispersions prepared without F-68 are shown in Figure 5a. The weight ratio of EC/PC-to-water was set to 25/75. The dispersion was opaque, and FF-TEM revealed non-uniform photoresist aggregates similar to secondary particles in the range of 50–150 nm. This aggregation was caused by interactions between the hydrophobic photoresist polymers. In contrast, the photoresist dispersion prepared with F-68 was transparent, which is consistent with the observation of single-photoresist particles by FF-TEM (Figure 5b). The Pluronic PEO chain extends into the bulk, and the PPO chain anchors strongly to the hydrophobic surface,^29^ thus preventing photoresist aggregation by F-68 adsorption on the photoresist particles.

SAXS measurements were performed to investigate the structure of the photoresist dispersions. Figure 5c shows the scattering intensity profiles for each system. We set the F-68 concentration to 10% w/w to increase the X-ray scattering intensity originating from the adsorbed F-68 on the photoresist. The photoresist dispersibility was verified to be independent of the F-68 concentration in the range of 1–10% w/w. The shoulder was detected around 0.1 nm^–1^ for photoresist aggregates without F-68. When F-68 was added, the band shifted to a wider angle than in the absence of F-68. We assumed that the band was caused by the complex structure formed between the photoresist particle and F-68 because the scattering data for the F-68 solution did not detect any characteristic intensity.

Figure 6a shows the PDDF profiles calculated by the IFT analysis of the scattering data of the photoresist dispersion. The *p*(*r*) represents the total value of the product of electron density fluctuations in a minute volume at both ends of an arbitrary distance, *r*, for all possible combinations of two points in the particle, and the curve provides information on the particle size and shape.^20^ The highest diameter of the photoresist particle without and with F-68 was calculated as 73.0 and 40.5 nm, estimated from the zero-convergence point, respectively; these sizes were correlated with the FF-TEM images. As expected, the curves were distinguishable depending on the presence or absence of F-68. Without F-68, the PDDF curve can be described as a bell-shape,^30^ suggesting a homogeneous electron density fluctuation inside the particle regardless of whether it is positive or negative. Conversely, the classic core-corona structure^30,31^ was formed for the curve in the presence of F-68. Hence, the complex of the photoresist and F-68 would have a hydrophobic photoresist as the core, and the PEO and PPO chains of F-68 as the corona and anchor parts, respectively. According to a previous study,^32^ F-68 forms micelles at 40% w/w or more at room temperature. In particular, although F-68 was dissolved as unimers, it possibly formed a core-corona complex with the photoresist in the EC/PC/water mixture. This hypothesis is also supported by the PDDF profile of the pure P-123 micelle system,^20^ which shows a bell-like shape owing to the similarities in the structures of PEO and PPO. Therefore, the electron density of the photoresist is suggested to be lower than that of PEO and PPO.

Figure 6b shows the electron density profiles calculated by the deconvolution analysis of the PDDF curves. In the absence of F-68, the profile gradually decreases to *r* = 0 and converges at approximately 40 nm, corresponding to the radius. Δρ(*r*) is positive because of the uniform internal electron density. In contrast, the electron density profile of the photoresist was lower than that of the solvent, and the negative electron density profile demonstrated the hydrophobic core of the complexes with F-68. As the distance from the center of the sphere increases, the profile passes through the zero point, which is defined as the length of the hydrophobic core. Subsequently, Δρ(*r*) becomes positive and converges to zero, which is determined as the length of the hydrophilic corona. The obtained thicknesses of the hydrophilic corona and the hydrophobic core were 11.0 and 11.8 nm, respectively. Considering that the thickness of the hydrophilic corona (11.0 nm) is shorter than the contour length of the PEO chain (28 nm),^12^ the PEO chains are expected to be folded within the corona. Despite the negative Δρ(*r*) of the photoresist in the complex with F-68, the photoresist particle without F-68 showed constantly positive value. This is because the PDDF curve showed positive *p*(*r*) even though the Δρ(*r*) was homogeneously negative inside the photoresist based on the definition of the *p*(*r*).

AFM analysis was performed to evaluate the interaction forces between the F-68 adsorption layers. Approaching force curves were obtained in an F-68 aqueous solution (1% w/w) and water after 10 cycles of water replacement (Figure 7). In both systems, the repulsive force increased continuously as the tip approached the substrate (Figure 7, inset). This repulsion is attributed to the adsorbed layers between the cantilever tip and the photoresist film because F-68 is assumed to be adsorbed onto the probe.^12,33^ The repulsive interactions remained even after repeated washing with water. In the absence of F-68, an attractive force was detected between the photoresist and the cantilever tip in water (Supporting Information, Figure S3).

For a discussion of the film properties, we analyzed the compression force curve data using the Milner–Witten–Cates (MWC) theory.^12,28,34^ Milner et al. describe the density distribution function of a polymer brush as a parabolic profile based on the self-consistent field theory. This model can be applied to the force curve using the Derjaguin approximation.^35,36^ We also performed symmetric measurements (both surfaces covered with F-68) and asymmetric measurements (one photoresist surface covered with F-68), corresponding to the theoretical formulas in eqs 9 and 11, respectively.^36^ This theory enables the estimation of the uncompressed brush thickness (*L*) and the average distance between grafting points (*s*) by fitting the experimental data.
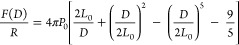
9
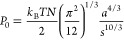
10
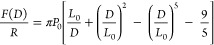
11where *L*_0_ is the equilibrium brush thickness (*L*_0_ is assumed to be 1.3*L*^19^), *D* is the surface separation distance, *R* is the radius of the cantilever tip (15 nm, nominal value), *k*_B_ is the Boltzmann’s constant, *T* is the absolute temperature, *N* is the number of segments in the polymer chain, and *a* is the segment length. We assigned the symmetric and asymmetric models to the F-68 aqueous and water-rinsing systems, respectively. This assumption is based on the QCM-D results, in which the F-68 on silica was almost completely desorbed by rinsing with water (Supporting Information, Figure S4).

Figure 7 also shows a good fit between the MWC model and the experimental data for each condition. This indicates that F-68 formed a brush layer on the photoresist and maintained its structure after rinsing with water. The uncompressed layer thickness (*L*) was 19.8 (in F-68 solution) and 17.3 nm (after rinsing with water), respectively. This similarity suggests that the PPO chain of F-68 was strongly adsorbed on the photoresist film. The brush thickness obtained from AFM (in water) was larger than the hydrophilic corona thickness (11.0 nm) calculated from the SAXS deconvolution profile. This variation was affected by the presence of the EC/PC mixture in the SAXS measurements. Generally, the polymer brush extends into the bulk solution because of its higher affinity for the solvent and larger adsorption amount.^37^ Considering that the EC/PC mixture is more solvophilic than water,^12^ the shorter thickness estimated by the SAXS measurements was probably caused by a decreased adsorption amount of F-68 in the presence of EC/PC in the solvent. However, the resolution and sensitivity of AFM and SAXS must be considered. The other fitted parameter, *s*, in the F-68 solution and water was estimated to be 6.3 and 2.8 nm, respectively. The dense packing after water rinsing may be attributed to the loose packing of F-68 adsorbed on the cantilever tip, resulting in the loss of resistance to compression.

## Conclusions

In this study, we characterized the adsorption/desorption behaviors of Pluronic F-68 and a photoresist on/from an ITO substrate to understand the anti-adsorption mechanism during the photoresist-stripping process. In addition, we investigated the dispersion of photoresist particles. The results were compared with and without F-68. QCM-D measurements revealed that the photoresist adsorbed rigidly on ITO in a mixed solution of EC and PC, and the amount of adsorbed photoresist further increased upon water rinsing. This residue could not be removed from the surface by repeated rinsing with EC/PC. In contrast, the adsorption amount of the photoresist decreased remarkably in the presence of F-68. This effect can be attributed to two factors. The first factor is the physical inhibition of the photoresist on the ITO substrate. Compared to bare ITO, the surface covered with F-68 featured the effect of impeding the adsorption rate of the photoresist. The second factor is the contribution of the photoresist particles to the dispersion stability. Hydrophobic photoresist polymers aggregated on the substrate and in the bulk solution when rinsed with water. However, SAXS and AFM measurements suggest that F-68 forms a core-corona structure with photoresist particles in the bulk and a brush structure on the photoresist film. These structures, in which the PEO chains extend into the bulk and the PPO chains are anchored to the photoresist, prevent aggregation between photoresist particles and are consistent with the FF-TEM images. Consequently, we proposed a mechanism to prevent photoresist adsorption with F-68, which could become a platform for many cleaning technologies as well as in the electronics industry.

## Supporting Information Available

SEM images of the photoresist aggregates after the QCM-D water rinsing process of the ITO substrate; approaching force curve data obtained in the EC/PC mixture and F-68 solution dissolved in the EC/PC mixture; approaching force curve data obtained in water against the photoresist film; and frequency and dissipation shifts as a function of time of the 5th, 7th, and 9th overtones on the bare silica substrate and the concentrations of F-68 in water were set at 0.1 mmol/L, 1 mmol/L, and 10 mmol/L (PDF)

## Abbreviations

1D: one-dimensional
AFM: atomic force microscopy
EC: ethylene carbonate
FF-TEM: freeze-fracture transmission electron microscopy
ITO: indium tin oxide
MWC: Milner–Witten–Cates
PC: propylene carbonate
PDDF: pair distance distribution function
PGMEA: propylene glycol monomethylethyl ether acetate
PEO: poly(ethylene oxide)
PPO: poly(propylene oxide)
QCM-D: quartz crystal microbalance with dissipation monitoring
SAXS: small-angle X-ray scattering
SEM: scanning electron microscopy
UV: ultraviolet
