# Supplementary material for: Anti-adsorption Mechanism of Photoresist by Pluronic Surfactants: An Insight into Their Adsorbed Structure
Source: Langmuir. 2023 May 20;39(22):7876–83. doi: 10.1021/acs.langmuir.3c00714 (PMC10249399; doi:10.1021/acs.langmuir.3c00714)
Supplement: Supplementary file 1 — la3c00714_si_001.pdf [file la3c00714_si_001.pdf]

Supporting Information

Anti-adsorption Mechanism of Photoresist by Pluronic Surfactants: An Insight into Their Adsorbed Structure

Masaki Hanzawa [a*], Taku Ogura [a,b], Koji Tsuchiya [b], Masaaki Akamatsu [c],
Kenichi Sakai [b,d*], and Hideki Sakai [b,d]

[a] NIKKOL GROUP Nikko Chemicals Co., Ltd. 3-24-3 Hasune, Itabashi, Tokyo 174-0046, Japan
[b] Research Institute for Science and Technology, Tokyo University of Science,
2641 Yamazaki, Noda, Chiba 278-8510, Japan
[c] Department of Chemistry and Biotechnology, Faculty of Engineering, Tottori University,
4-101 Koyama-Minami, Tottori 680-8552, Japan
[d] Department of Pure and Applied Chemistry, Faculty of Science and Technology,
Tokyo University of Science, 2641 Yamazaki, Noda, Chiba 278-8510, Japan

Corresponding authors: hanmasa@nikkolgroup.com, k-sakai@rs.tus.ac.jp

Content is 5 pages including 4 figures.

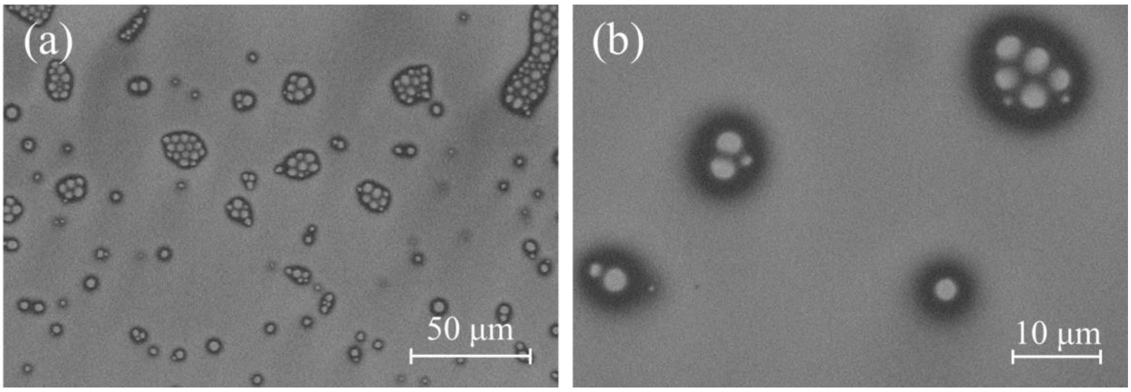

Figure S1. Scanning electron microscopy (SEM) images of the photoresist aggregates after the QCM-D water rinsing process of the ITO substrate (Figure 1); (a) 500-fold and (b) 2,000-fold magnification. The SEM analysis was performed using a Hitachi High-Tech TM 4000Plus instrument. The acceleration voltage was set to 5 kV.

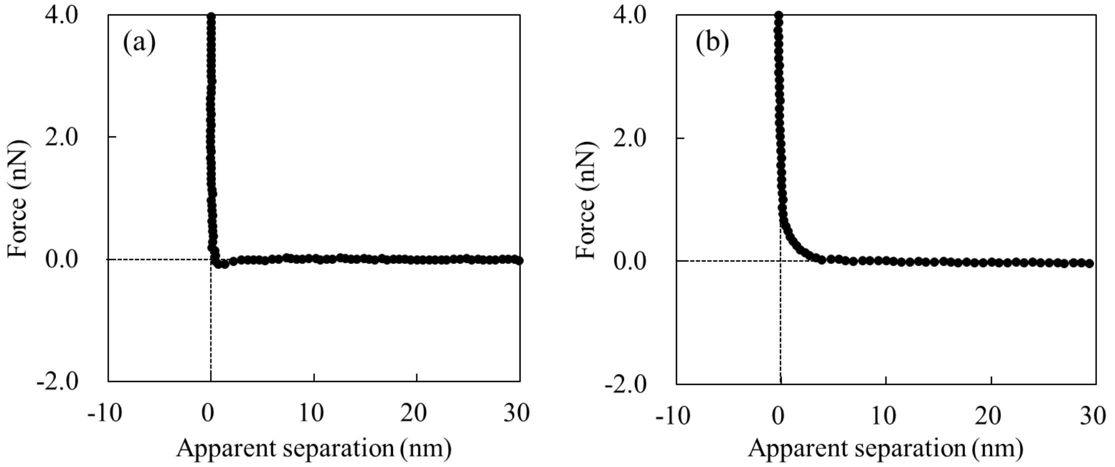

Figure S2. Approaching force curve data obtained in (a) the EC/PC mixture and (b) F-68 (1% w/w) solution dissolved in the EC/PC mixture. The solid substrate used here was ITO.

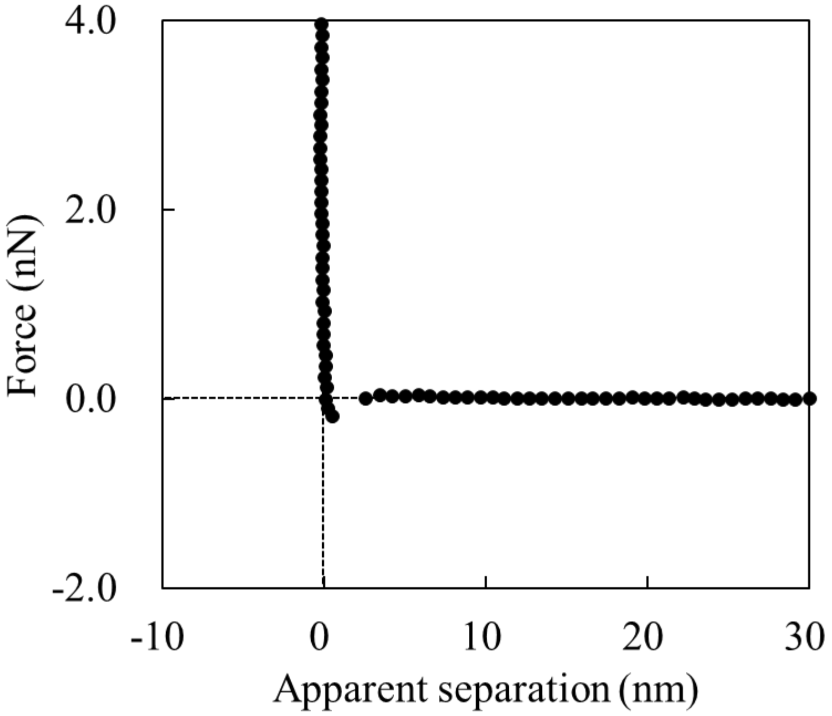

Figure S3. Approaching force curve data obtained in water against the photoresist film.

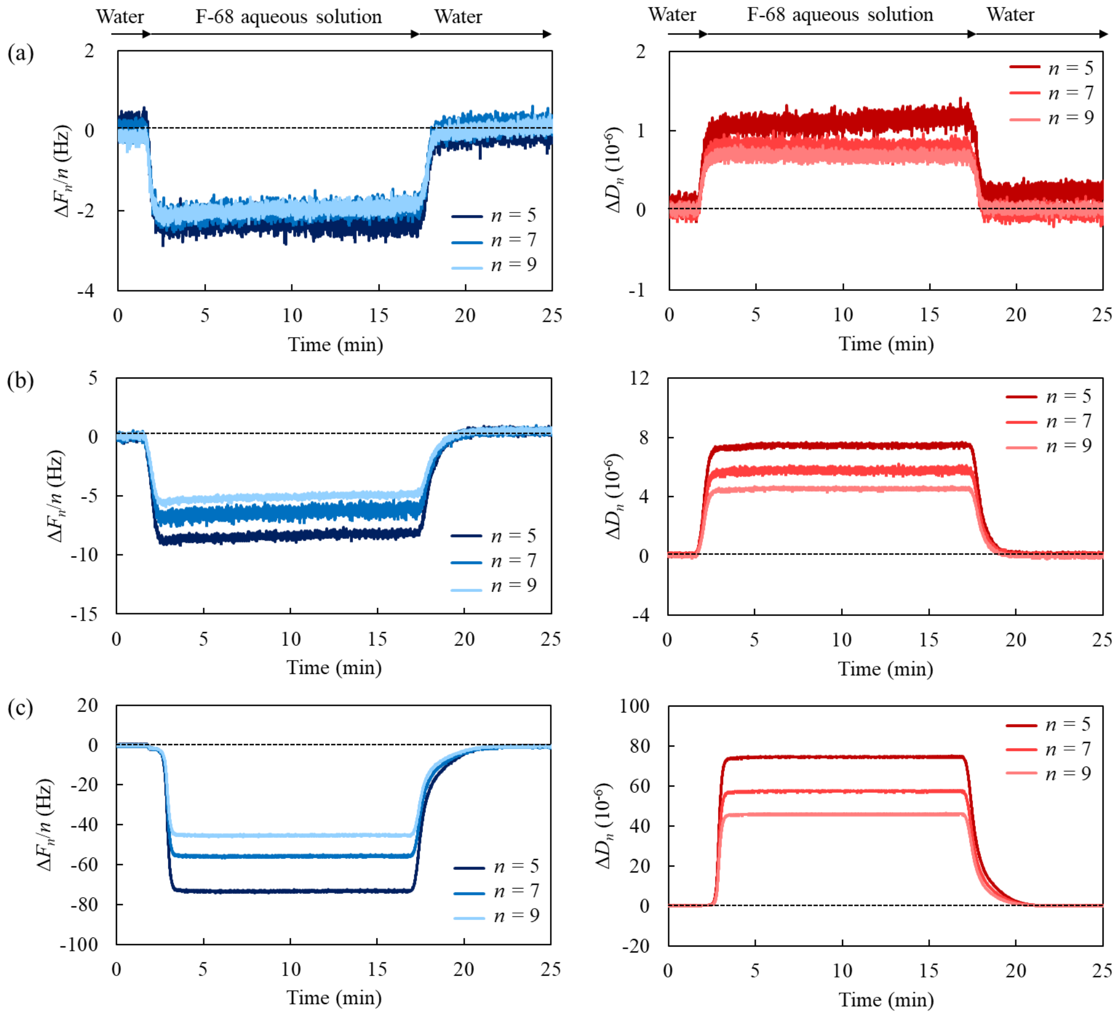

Figure S4. Frequency (left) and dissipation (right) shifts as a function of time of the 5th, 7th, and 9th overtones on the bare silica substrate. The concentrations of F-68 in water were set at (a) 0.1 mmol/L (0.08% w/w), (b) 1 mmol/L (0.8% w/w), and (c) 10 mmol/L (8% w/w)).
